# P-1354. Carbapenemase Gene Detection in Clinically Significant *Pseudomonas aeruginosa* Isolates from A Tertiary Care Health Center: A Prospective Study

**DOI:** 10.1093/ofid/ofae631.1531

**Published:** 2025-01-29

**Authors:** Deepak Kumar, Monika Chaudhary, Ravisekhar Gadepalli, meruvu Hari Vaishnavi, alisha aggarwal, Vibhor Tak, Gopal Krishna Bohra, Durga Shankar Meena, Naresh Kumar Midha, Subhashree Samantaray, Hitendra Pal Solanki, Nikhil Kothari, Pradeep Bhatia, M K Garg

**Affiliations:** All India Institute of Medical Sciences, Jodhpur (India), Jodhpur, Rajasthan, India; AIIMS, JODHPUR, JODHPUR, Rajasthan, India; AIIMS, JODHPUR, JODHPUR, Rajasthan, India; AIIMS, JODHPUR, JODHPUR, Rajasthan, India; AIIMS, JODHPUR, JODHPUR, Rajasthan, India; AIIMS Jodhpur, Jodhpur, Rajasthan, India; AIIMS, Jodhpur, Rajasthan, India; AIIMS, Jodhpur, Rajasthan, India; AIIMS, Jodhpur, Rajasthan, India; AIIMS JODHPUR, Jodhpur, Rajasthan, India; AIIMS, JODHPUR, JODHPUR, Rajasthan, India; AIIMS, JODHPUR, JODHPUR, Rajasthan, India; AIIMS, JODHPUR, JODHPUR, Rajasthan, India; AIIMS, Jodhpur, Rajasthan, India

## Abstract

**Background:**

*Pseudomonas aeruginosa* is a leading pathogen causing widespread hospital acquired infections worldwide. As WHO has also designated Carbapenem resistant *Pseudomonas aeruginosa* as one of three Critical Priority Pathogen, we decided to conduct a study on the *Pseudomonas aeruginosa* isolates to find out the presence of carbapenemase genes using RT-PCR and to find out the relation between the phenotypic assessment of carbapenem resistance with genotypic detection of carbapenemase genes.

**Figure:**
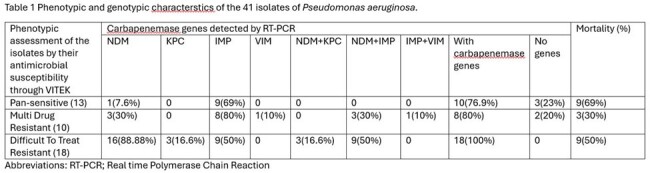

**Methods:**

Total 41 patients were included in this prospective observational study done for 6 months from 1^st^ July 2023 to 31^st^ December 2023. *Pseudomonas aeruginosa* isolates from the patients with age >18 years, ICU admission for more than 48 hours and absence of any coinfection were included and those patients with polymicrobial infections and multisite infections were excluded. Isolates were obtained from the blood, respiratory and pyogenic samples and antimicrobial susceptibility was done by VITEK for various antimicrobials and broth microdilution for colistin. The bacterial colony was used for DNA extraction using Bacterial Genomic DNA Extraction Kit in accordance with the manufacturer’s instruction. Multiplex RT PCR was done to detect genes blaVIM, blaNDM, blaKPC and blaIMP.

**Figure:**
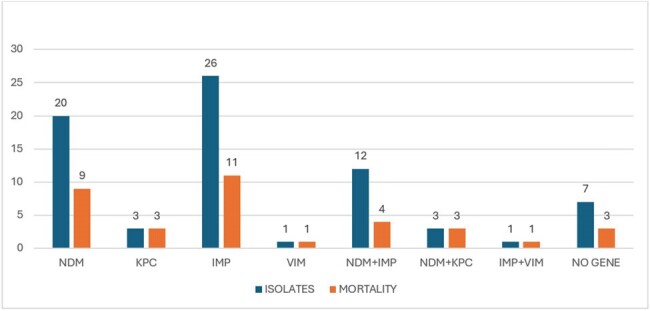

**Results:**

Out of 41 isolates, as depicted in figure 1, blaIMP was most common gene found in 26(63.41%), followed by blaNDM in 20(48.7%) isolates. Combination of blaNDM and blaIMP was found in 12 (29.2%) and blaNDM with blaKPC in 3(7.3%) patients. As shown in table 1 even in isolates which were found pan sensitive to all antimicrobials in VITEK were also harboring genes for carbapenemase. blaIMP was found in 9(69%) of these isolates out of 13 pan sensitive ones. Attributable mortality according to phenotypic resistance is described in table 1. Figure 2 is amplification plot of blaNDM by RT PCR of one of the isolates.

**Figure:**
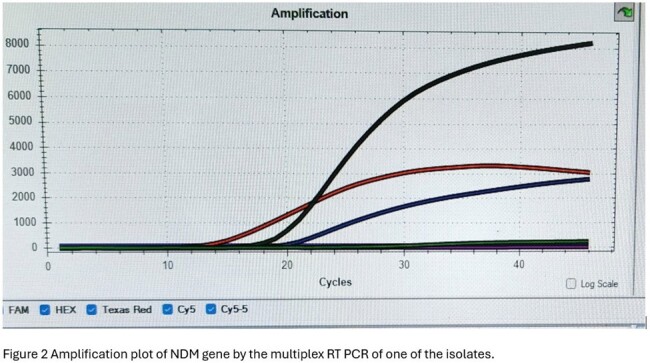

**Conclusion:**

We found presence of blaNDM and blaIMP genes in our isolates of *Pseudomonas aeruginosa* as compared to global presence of blaKPC and blaVIM. High prevalence of carbapenemase genes even in pan-sensitive isolates can be attributed to excessive use of carbapenems and larger studies are required to find out its impact on clinical outcome.

**Disclosures:**

**All Authors**: No reported disclosures

